# LncRNA *lncLLM* Facilitates Lipid Deposition by Promoting the Ubiquitination of MYH9 in Chicken LMH Cells

**DOI:** 10.3390/ijms251910316

**Published:** 2024-09-25

**Authors:** Qi-Hui Jia, Yu-Zhu Cao, Yu-Xin Xing, Hong-Bo Guan, Cheng-Lin Ma, Xin Li, Wei-Hua Tian, Zhuan-Jian Li, Ya-Dong Tian, Guo-Xi Li, Rui-Rui Jiang, Xiang-Tao Kang, Xiao-Jun Liu, Hong Li

**Affiliations:** 1 College of Animal Science and Technology, Henan Agricultural University, Zhengzhou 450046, China; jqh2021@stu.henau.edu.cn (Q.-H.J.); cyz2021@stu.henau.edu.cn (Y.-Z.C.); xyx2022@stu.henau.edu.cn (Y.-X.X.); ghb2023@stu.henau.edu.cn (H.-B.G.); mcl2023@stu.henau.edu.cn (C.-L.M.); 18848897291@163.com (X.L.); tianweihua@henau.edu.cn (W.-H.T.); lizhuanjian@163.com (Z.-J.L.); tianyadong@henau.edu.cn (Y.-D.T.); guoxili2023@henau.edu.cn (G.-X.L.); jrrcaas@163.com (R.-R.J.); xtkang2023@henau.edu.cn (X.-T.K.); 2 Key Laboratory of Livestock and Poultry Resources (Poultry) Evaluation and Utilization, Ministry of Agriculture and Rural Affairs, Henan Agricultural University, Zhengzhou 450046, China; 3 International Joint Research Laboratory for Poultry Breeding of Henan, Henan Agricultural University, Zhengzhou 450046, China

**Keywords:** lncRNA, lipid metabolism, MYH9, fatty liver, ubiquitination

## Abstract

The liver plays an important role in regulating lipid metabolism in animals. This study investigated the function and mechanism of *lncLLM* in liver lipid metabolism in hens at the peak of egg production. The effect of *lncLLM* on intracellular lipid content in LMH cells was evaluated by qPCR, Oil Red O staining, and detection of triglyceride (TG) and cholesterol (TC) content. The interaction between *lncLLM* and MYH9 was confirmed by RNA purification chromatin fractionation (CHIRP) and RNA immunoprecipitation (RIP) analysis. The results showed that *lncLLM* increased the intracellular content of TG and TC and promoted the expression of genes related to lipid synthesis. It was further found that *lncLLM* had a negative regulatory effect on the expression level of MYH9 protein in LMH cells. The intracellular TG and TC content of MYH9 knockdown cells increased, and the expression of genes related to lipid decomposition was significantly reduced. In addition, this study confirmed that the role of *lncLLM* is at least partly through mediating the ubiquitination of MYH9 protein to accelerate the degradation of MYH9 protein. This discovery provides a new molecular target for improving egg-laying performance in hens and treating fatty liver disease in humans.

## 1. Introduction

The liver plays a central role in lipid metabolism and is involved in various biochemical processes that maintain lipid homeostasis [1]. These processes include de novo synthesis of fatty acids, the uptake of circulating fatty acids, and fatty acid oxidation [2]. The liver has the ability to convert excess energy substrates into triglycerides (TG) for storage in hepatocytes or release into the bloodstream [3]. Additionally, the liver is an important site for the synthesis of cholesterol (TC) and lipoproteins. Lipoproteins, such as low-density lipoprotein (LDL) and high-density lipoprotein (HDL), transport and distribute TC and TG in the bloodstream, thereby regulating lipid balance [4]. The TC synthesized in the liver serves as a precursor for sex hormones and glucocorticoids, playing a crucial role in growth and development [5]. Furthermore, the liver can re-esterify excess intracellular fatty acids into TG, thereby reducing the cytotoxicity of fatty acids [6].

Long noncoding RNAs (LncRNAs) are emerging as a family of gene regulators [7]. LncRNA participates in the regulation of gene expression and biological processes at the transcription level, post-transcriptional level, and interactions with proteins through multiple mechanisms [8,9,10]. Recent studies have reported that lncRNA plays a role in mediating the process of protein ubiquitination [11,12]. More and more studies have shown that lncRNA is involved in the lipid metabolism process. For example, lncRNA-HC acts as a molecular sponge to counteract the inhibitory effect of *miR-130b-3p* on *PPARG* [13]. *lncLSTR* regulates the FXR/apoC2 pathway by modulating the TDP-43/Cyp8b1 axis, thereby affecting TG clearance rates [14]. *lncHLEF* promotes hepatic lipid synthesis in chickens through the miR-2188-3p/GATA6 axis and encoded peptides [15].

The *MYH9* gene encodes the heavy chain of non-muscle myosin of class II, isoform A (NMIIA). *MYH9* has been reported to be involved in a variety of biological processes [16,17,18]. Recently, *MYH9* has been reported to play a central role as a skeleton protein in intracellular lipid droplet metabolism [19,20].

The meat of Lushi blue shell chicken, a native Chinese chicken, is tender, and its eggs are high in protein and low in cholesterol. Interestingly, in our previous study, we found that *lncLLM* expression was significantly higher during the peak egg-laying period than during the pre-laying period [15]. One of the most obvious changes in poultry during the peak laying period is the vigorous lipid metabolism process in the liver. However, it is unclear whether *lncLLM* is involved in regulating lipid metabolism in chicken liver. The LMH cell line was established from chicken primary hepatocellular carcinoma cells, and is commonly used to explore the lipid metabolism regulation mechanism in chicken liver. Therefore, in this study, LMH cells were used to explore the function and regulation mechanism of *lncLLM* in chicken liver lipid metabolism.

## 2. Results

### 2.1. Expression Pattern, Coding Ability Prediction and Subcellular Localization of lncLLM

**Figure 1:**
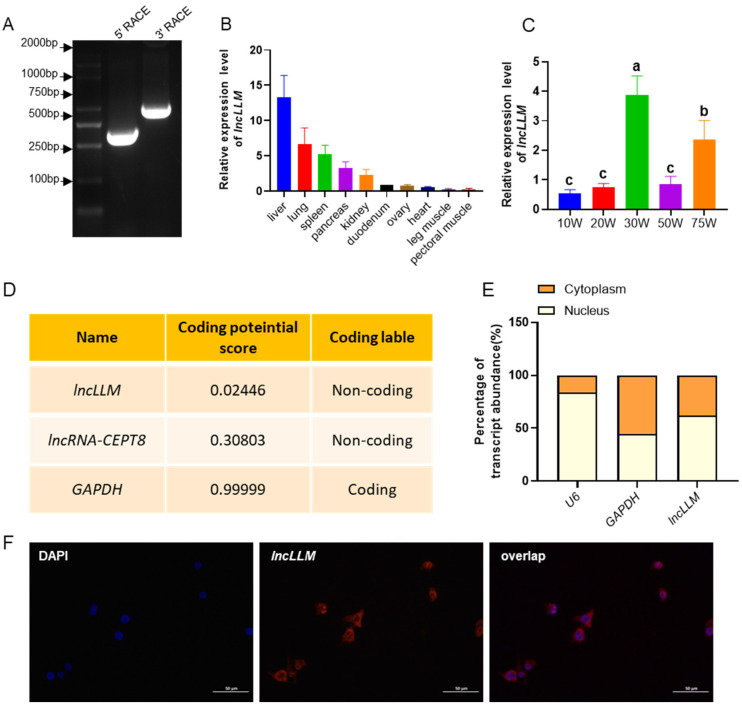
Expression pattern, coding ability prediction and subcellular localization of *lncLLM*. (**A**) Rapid amplification of cDNA ends (RACE) for the 5′ end and 3′ end was performed to determine the full length of *lncLLM*. (**B**) qPCR analysis of the expression of the *lncLLM* in different tissues of 30 w hens (*n* = 6). W means ages of weeks (**C**) qPCR analysis of the expression of the *lncLLM* in liver tissue at different stages (*n* = 6). Different lowercase letters in each bar denote a significant difference (*p* < 0.05). (**D**) Coding potential score of *lncLLM*, *lncRNA-CEPT8* and *GAPDH* by CPC program. (**E**) qPCR analysis of *lncLLM* expression in cytoplasm and nuclear fractions of LMH cells (*n* = 3). (**F**) The distribution of *lncLLM* in the cytoplasm and nuclei of LMH cells by FISH assay. Cell nuclei were stained with DAPI (blue), and *lncLLM* was hybridized with *lncLLM* probe (red), Scale bar 50 μm.

The full length of *lncLLM* is 1441 bp, which was confirmed by RACE assay (Figure 1A). The qPCR results showed that *lncLLM* had the highest expression level in the liver, followed by the lung, spleen, pancreas, duodenum, and relatively low expression levels in the heart, pectoral muscle, and leg muscle (Figure 1B). The spatiotemporal expression analysis results showed that the expression level of *lncLLM* in the liver of chickens at 30 weeks was significantly higher than that at other stages (*p* < 0.05). The expression of *lncLLM* in the liver of chickens at 75 weeks was significantly higher than that at other stages except 30 weeks (*p* < 0.05) (Figure 1C). Coding Potential Calculator (CPC) calculation results indicated that *lncLLM* has non-coding potential, similar to the reported *lncRNA-CEPT8* (Figure 1D) [21]. The expression of *lncLLM* was detected in both the cytoplasm and nucleus of liver cells by nuclear and cytoplasmic qPCR as well as FISH analysis (Figure 1E,F).

### 2.2. LncLLM Modulating Lipid Metabolism in LMH Cells

**Figure 2:**
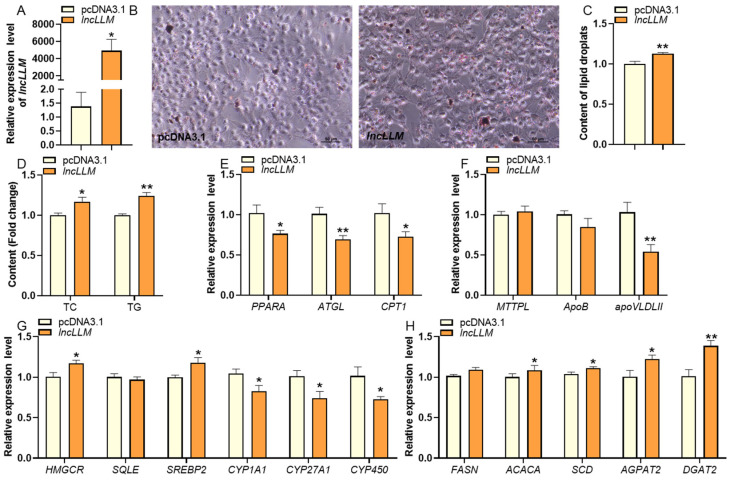
Effects of overexpressing *lncLLM* on TG, TC contents, and the expression of related genes. (**A**) qPCR analysis of expression of the *lncLLM* after transfection *lncLLM* (*n* ≥ 4). (**B**,**C**) Oil red o staining analysis of lipid droplet content in the control and *lncLLM*-overexpressed LMH cells (*n* = 3). (**D**) Detection of TC and TG content in the control and *lncLLM*-overexpressed LMH cells (*n* ≥ 4). (**E**–**H**) The relative mRNA expression levels of lipid-related genes in the control and *lncLLM*-overexpressed LMH cells via qPCR (*n* ≥ 4). * means 0.01 < *p* < 0.05, ** *p* < 0.01.

To explore the biological function of *lncLLM* in chicken liver lipid metabolism process, *lncLLM* over-expression and siRNA experiments were conducted in chicken LMH cells. *lncLLM* mRNA expression level shows approximately a 3000-fold increase in overexpressed LMH cells when compared to the control cells (Figure 2A). Interestingly, compared to the negative control group, the number and size of intracellular lipid droplets in *lncLLM* overexpressed LMH cells showed a significant increase (Figure 2B,C), as well as a significant increase in the contents of intracellular TC and TG (*p* < 0.05) (Figure 2D). The results demonstrated that the *lncLLM* overexpressed group exhibited significant upregulation of TG synthesis-related genes, including acetyl-CoA carboxylase alpha (*ACACA*), stearoyl-CoA desaturase (*SCD*), 1-acylglycerol-3-phosphate O-acyltransferase 2 (*AGPAT2*), Diacylglycerol O-Acyltransferase 2 (*DGAT2*), as well as cholesterol synthesis genes such as 3-hydroxy-3-methylglutaryl-CoA reductase (*HMGCR*), squalene epoxidase (*SQLE*), and sterol regulatory element binding protein 2 (*SREBP2*). Additionally, a lipid transport-related gene, very-low-density apolipoprotein II (*APOVLDLII*), was also significantly activated in the *lncLLM* overexpressed group (*p* < 0.05). However, genes involved in TG degradation progress, such as peroxisome proliferator activated receptor alpha (*PPARA*), adipose triglyceride lipase *(ATGL)*, carnitine palmitoyl transferase 1 (*CPT1*), and cholesterol degradation genes Cytochrome P450 Family 1 Subfamily A Member 1 (*CYP1A1*), Cytochrome P450 Family 27 Subfamily A Member 1 (*CYP27A1*), Cytochrome P450 Enzymes (*CYP450*), were significantly inhibited (*p* < 0.05) (Figure 2E–H).

**Figure 3:**
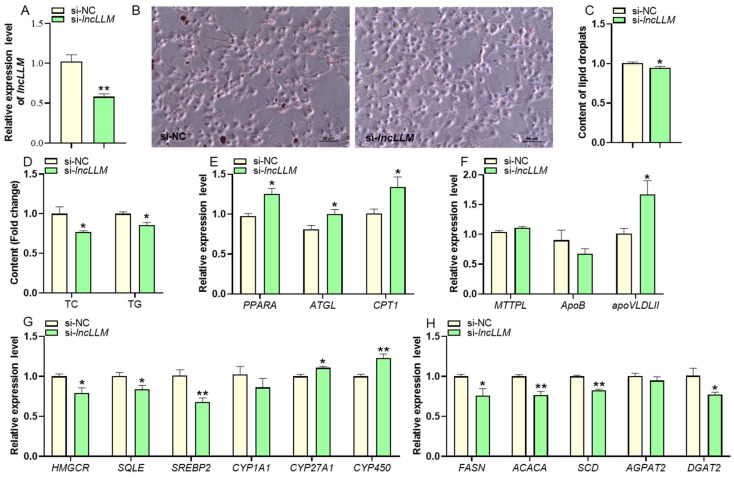
Effects of knockdown of *lncLLM* on TG, TC contents, and the expression of related genes. (**A**) qPCR analysis of expression of the *lncLLM* after transfection si-*lncLLM* (*n* ≥ 4). (**B**,**C**) Oil red o staining analysis of lipid droplet content in the control and *lncLLM* knockdown LMH cells (*n* = 3). (**D**) Detection of TC and TG content in the control and *lncLLM* knockdown LMH cells (*n* ≥ 4). (**E**–**H**) The relative mRNA expression levels of lipid-related genes in the control and *lncLLM* knockdown LMH cells via qPCR (*n* ≥ 4). * means 0.01 < *p* < 0.05, ** *p* < 0.01.

In contrast, *lncLLM* expression is reduced by approximately 50% in the si-*lncLLM* knockdown LMH cells as compared to control cells (Figure 3A). Compared to the siRNA negative control group, a significant reduction in both the number and volume of intracellular lipid droplets in *lncLLM* knocked down LMH cells was observed (Figure 3B,C), as well as the intracellular TC and TG contents (Figure 3D). Accordingly, the expression levels of the aforementioned-gene associated with lipid metabolism exhibited an inverse expression trend to that observed in the *lncLLM* overexpression experiment (Figure 3E–H).

### 2.3. Endogenous Protein MYH9 Interacted with lncLLM in LMH Cells

**Figure 4:**
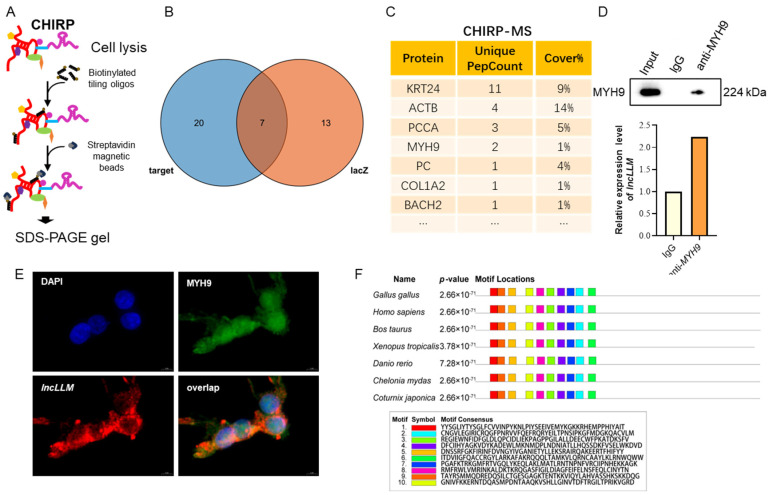
Identification of endogenous protein partners associated with *lncLLM* in LMH cells. (**A**) Schematic of CHIRP-MS assay. (**B**) Venn diagram of negative probe (lacZ) and *lncLLM* probe (target) pull-down protein. (**C**) The partial list of proteins identified by MS. (**D**) WB analysis of MYH9 protein after IP with MYH9 antibody. qRT-PCR detection of *lncLLM*. (**E**) The co-localization of MYH9 and *lncLLM* by FISH coupled with IF. (**F**) Conservative motifs analysis of MYH9 among species. Rectangles of different colors represent different motifs, and the length of the rectangle represents the number of amino acids.

To investigate the action mechanism of *lncLLM* in regulating the lipid metabolism of LMH cells, we performed a comprehensive identification of RNA-binding proteins by mass spectrometry assay (Figure 4A). In total, we identified 20 specific potential protein partners of *lncLLM* (Figure 4B,C). Based on the RNA-protein interaction prediction, MYH9 was selected to be the candidate interaction protein of *lncLLM*.

The RNA binding protein immunoprecipitation (RIP) assay demonstrated that *lncLLM* could interact with MYH9 *lncLLM*. As expected, *lncLLM* was enriched in the immunoprecipitation complexes with the addition of MYH9 protein antibody (Figure 4D). In addition, the results of FISH for *lncLLM* and immunofluorescence staining for MYH9 protein showed their co-localization in the cytoplasm (Figure 4E). By comparing the amino acid sequences of MYH9 between species, it was found that they have the same motif among species, which indicates that MYH9 may be involved in the liver lipid metabolism process (Figure 4F).

### 2.4. Effect of MYH9 on TG and TC Contents in LMH Cells

**Figure 5:**
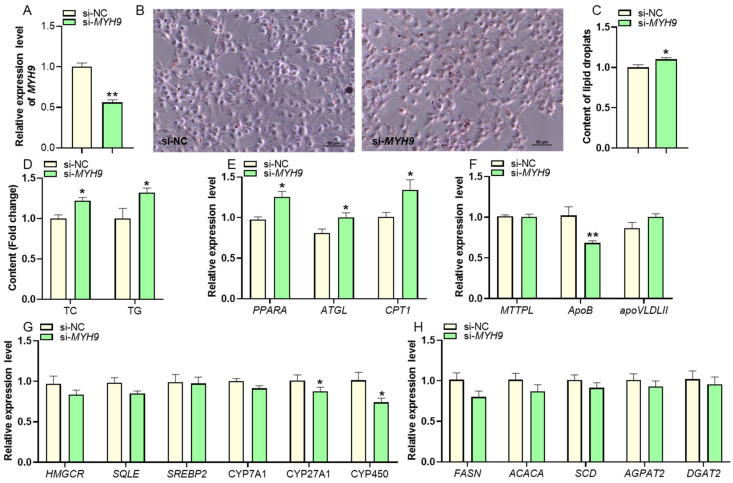
Effect of *MYH9* on lipid accumulation in LMH cells. (**A**) qPCR analysis of expression of the *MYH9* after transfection si-*MYH9* (*n* ≥ 4). (**B**,**C**) Oil red staining analysis of lipid droplet content in the control and *MYH9*-knockdowned LMH cells (*n* = 3). (**D**) Detection of TC and TG content in the control and *MYH9* knockdown LMH cells (*n* ≥ 4) (**E**–**H**) The relative mRNA expression levels of lipid-related genes in the control and *MYH9*-knockdowned LMH cells via qPCR (*n* ≥ 4). * means 0.01 < *p* < 0.05, ** *p* < 0.01.

To evaluate the biological role of *MYH9* in lipid metabolism, a knockdown experiment was performed in LMH cells. *MYH9* expression is reduced by approximately 50% in the si-*MYH9* knockdown LMH cells as compared to control cells (Figure 5A). Interestingly, compared to the negative control group, the number and size of intracellular lipid droplets in *si-MYH9* knockdown LMH cells showed a significant increase (*p* < 0.05) (Figure 5B,C), as well as a significant increase in the contents of intracellular TC and TG (*p* < 0.05) (Figure 5D). Interestingly, when we detected the above-mentioned lipid metabolism genes, compared to the control group, there was no significant change in the expression of genes related to TG and TC synthesis in MYH9 knockdown LMH cells, but the expression levels of TG degradation-related genes *PPARA, ATGL,* and *CPT1* and TC degradation-related genes *CYP27A1* and *CYP450* significantly decreased (*p* < 0.05) (Figure 5E–H).

### 2.5. lncLLM Controlling the Expression of MYH9 through Affecting Protein Ubiquitination

**Figure 6:**
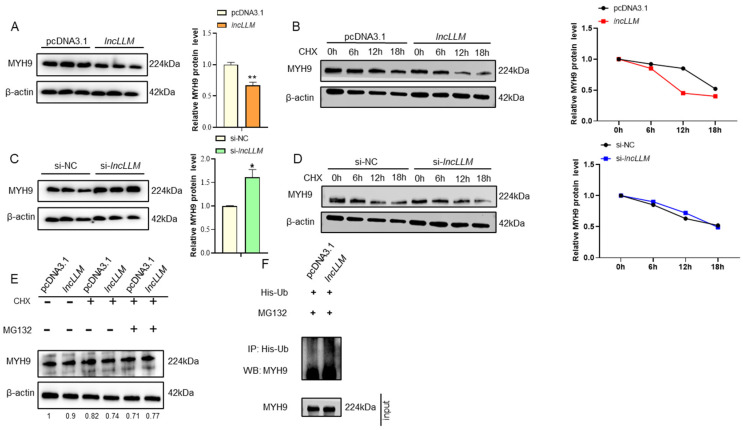
Effect of *MYH9* on lipid accumulation in LMH cells. (**A**) Detection of MYH9 protein expression in control and *lncLLM*-overexpressed LMH cells. (**B**) MYH9 in LMH cells transfected with pcDNA3.1 or *lncLLM* and treated with CHX for the indicated time. The degradation rates of MYH9 are shown on the right. (**C**) Detection of MYH9 protein expression in control and *lncLLM*-knockdowned LMH cells. (**D**) MYH9 in LMH cells transfected with si-NC or si-*lncLLM* and treated with CHX for the indicated time. The degradation rates of MYH9 are shown on the right. (**E**) MYH9 levels in cells treated with CHX or/and MG132 for 12 h. (**F**) WB analysis of MYH9 ubiquitination after IP with His antibody in cells transfected with His-Ub expressing plasmids. * means 0.01 < *p* < 0.05, ** *p* < 0.01.

To investigate the regulatory mechanism of *lncLLM* on MYH9 protein expression, we conducted overexpression or knockdown experiments of *lncLLM* in LMH cells to evaluate the levels of MYH9 protein. The results showed that the protein abundance of MYH9 in LMH cells overexpressing *lncLLM* was reduced by approximately 35% (Figure 6A). However, the protein abundance of MYH9 in LMH cells with *lncLLM* knockdown increased by approximately 50% (Figure 6C). To further confirm that *lncLLM* regulates the stability of MYH9 protein, we treated LMH cells with the protein synthesis inhibitor CHX. The results showed that MYH9 protein levels in both groups gradually decreased after 6 h, but the levels in the *lncLLM* overexpression group were lower than those in the control group at the same time (Figure 6B). In LMH cells with *lncLLM* knockdown, the levels of MYH9 were higher than those in the control group at the same time (Figure 6D). These results indicate that the stability of MYH9 is reduced when *lncLLM* is overexpressed. Interestingly, the abundance of MYH9 protein increased following MG132 treatment in LMH cells overexpressing *lncLLM* compared with the control group (Figure 6E). These results suggest that *lncLLM* may be involved in regulating the ubiquitination of MYH9.

To investigate whether *lncLLM* affects the ubiquitination of MYH9, we used MG132 to treat LMH cells with *lncLLM* overexpression for ubiquitination experiments. The results showed that the extent of MYH9 ubiquitination notably increased in *lncLLM* overexpression LMH cells (Figure 6F). The results further demonstrate that *lncLLM* can promote protein ubiquitination and degradation of MYH9.

## 3. Discussion

With the advancement of sequencing technology and the deepening of research, more and more lncRNAs have been proven to be involved in various biological processes, and have become known as important regulators of many important biological processes [22,23]. In recent years, there has been an increasing number of studies on lncRNA regulating hepatic lipid metabolism [24]. In humans, *lncRHPL* regulates hepatic VLDL secretion through the *hnRNPU*/*BMAL*/*MTTP* axis [25]. Another study showed that lncRNA MEG3 regulates hepatic lipogenesis by competitively binding with LRP6 to miR-21 [26]. *lncLLM* is highly expressed during the peak egg production period. We speculate that lncLLM regulates liver lipid production. Overexpression of *lncLLM* in LMH cells leads to a significant increase in intracellular TG and TC content, while interfering with *lncLLM* results in intracellular TG. TC content was significantly reduced, indicating that *lncLLM* indeed contributes to hepatic lipid production. In addition, *lncLLM* is poorly conserved, and we speculate that this may be caused by the differences in physiological structure between poultry and mammals.

As gene regulatory molecules, the specific cellular localization of lncRNA determines its biological function and mechanism of action [27]. Nuclear lncRNAs usually form complexes with RNA-binding proteins to exert their functions in regulating transcription. lncRNA HOTAIR binds to PRC2 and regulates the distribution of H3K27me3 in genomic targets in mammalian cells [28,29]. LncRNAs in the cytoplasm can also form complexes with proteins to affect cytoplasmic events at the post-transcriptional level, such as regulating protein localization and turnover, mRNA stability, and ubiquitination and degradation of target proteins [29,30,31]. Here, we identified a chicken-specific lncRNA, *lncLLM*, that is distributed in both the nucleus and cytoplasm, indicating that it may have multiple mechanisms of action. We identified the specific protein MYH9 that interacts with *lncLLM*. MYH9 is a component of the cytoskeleton. Studies have shown that MYH9 mediates the movement of intracellular lipid droplets and increases the contact between lipid droplets and mitochondria, thereby mediating the degradation of intracellular lipid droplets. Another study showed that MYH9 depletion leads to increased TG storage through impaired lipolysis [19,20,32]. Signals generated by mature adipocytes converge on progenitor cells to regulate the MYH9 protein and attenuate the rate of adipogenesis in the body [33]. Therefore, we believe that MYH9 is involved in regulating lipid levels in LMH cells by regulating the lipolysis process. HMBA ameliorates obesity by MYH9- and ACTG1-dependent regulation of hypothalamic neuropeptides [34]. Mitochondrial dynamic balance is regulated by molecular motors composed of myosin and actin cytoskeletal proteins. When the interaction between HSPA9 and MYH9 proteins is disrupted, it will lead to an imbalance in cytoskeleton-dependent three-dimensional dynamics [35].

The ubiquitin-proteasome pathway is one of the main pathways for protein degradation in cells. Cytoplasmic lncRNA can affect protein expression programs through the ubiquitin-proteasome pathway [36]. *LncRNA LYPAL1-AS1* regulates DSP protein stability through proteasomal degradation [37]. *lncRNA TPRG1-AS1* promotes the degradation of MYH9 in HASMC cells through the ubiquitin-proteasome pathway, inhibits the migration of HASMCs, and alleviates atherosclerosis in mice [38]. In bovine preadipocytes, lncBlncAD1 promotes adipocyte differentiation by mediating MYH10 protein ubiquitination [39]. We found that *lncLLM* co-localizes with MYH9 in the cytoplasm and may promote the degradation of MYH9 protein through the proteasome pathway; however, the specific mechanism requires further study.

The liver is generally considered to be the regulatory center for lipid metabolism and the primary site for de novo synthesis of fatty acids [40]. Elevated levels of FFAs and excessive accumulation of fat in the liver can lead to the development of nonalcoholic fatty liver disease and hepatic steatosis [41]. Lipid droplets (LDs) are cytoplasmic organelles for lipid storage that are surrounded by a phospholipid monolayer and coated with proteins involved in lipid metabolism [42,43]. Accumulation of lipid droplets in the liver is a hallmark of NAFLD [44,45]. Our study analyzes the conservation of MYH9 across species and elucidates the function of MYH9 in LMH cells, providing potential insights and molecular targets for fatty liver treatment.

## 4. Materials and Methods

### 4.1. Ethics Approval and Consent to Participate

The animal experiments were performed according to the Guide for the Care and Use of Laboratory Animals (Ministry of Science and Technology, Beijing, China, 2004). The study proposal was approved by the Institutional Animal Care and Use Committee (IACUC) of Henan Agricultural University (approval number: 11–0085). All methods were carried out in accordance with relevant guidelines and regulations. The study was carried out in compliance with the ARRIVE guidelines.

### 4.2. Sample Collection

Chinese local breed Lushi blue-shell hens were used as the experimental animal. Liver samples of Lushi blue-shell hens at the age of 10, 20, 30, 50, and 75-week-old were collected and stored at −80 °C after liquid nitrogen freezing, with six individuals for each time point. Tissue samples including heart, liver, spleen, lung, kidney, duodenum, pancreas, ovary, and pectoral muscles of Lushi blue-shell hens at the age of 20-week-old were collected and stored at −80 °C after liquid nitrogen freezing.

### 4.3. RNA Extraction, Reverse Transcription (RT), and Quantitative RT-PCR (qRT-PCR)

Total RNA from cells and tissues was extracted using Trizol reagent (Vazyme, Nanjing, China) according to the manufacturer’s protocol. RNA integrity and quality were detected using 1% agarose gel electrophoresis and NanoDrop2000 (Thermo Scientific, Wilmington, DE, USA), respectively. Subsequently, 1 μg of total RNA was reverse transcribed into cDNA according to the manufacturer’s instructions (Vazyme, Nanjing, China). The ChamQ Blue Universal SYBR qPCR Master Mix Kit (Vazyme, Nanjing, China) was used for cDNA quantification, according to the manufacturer’s protocol, in a LightCycler^®^ 96 Real-Time Detection instrument. The chicken glyceraldehyde-3-phosphate dehydrogenase (GAPDH) gene was used as an internal control. Data analysis was carried out using the comparative 2^−ΔΔCT^ method [46]. The primers information is shown in Supplementary Table S1.

### 4.4. Rapid-Amplification of cDNA Ends (RACE)

Liver tissues total RNA was used as RACE PCR template. The full length of *lncLLM* containing the transcriptional initiation and termination sites was confirmed by 5′-and 3′-RACE using the SMARTer RACE 5′/3′ Kit (Takara, Shiga, Japan, 634858). The products of the RACE PCR were cloned into the pCE2 TA/Blunt-Zero vector (Vazyme, Nanjing, China, C601) and sequenced by Tsingke Biotech (Beijing, China) [47]. The primers information was list in Supplementary Table S2.

### 4.5. Subcellular Fractionation

LMH cells were harvested to isolate nuclear and cytoplasmic fractions using the Cytoplasmic and Nuclear RNA Purification kit (Norgen, Belmont, CA, USA) according to the manufacturer’s instruction. The RNA extraction, cDNA synthesis, and qPCR were performed as described above. The U6 small nuclear RNA (*U6*) gene and *GAPDH* gene were used as nuclear and cytoplasmic marker genes, respectively.

### 4.6. Bioinformatics Analysis

The amino acid sequences of MYH9 different species including *Gallus gallus* (NP_990808.2), *Homo sapiens* (NP_002464.1), *Bos taurus* (NP_001179691.2), *Xenopus tropicalis* (XP_031756919.1), *Danio rerio* (NP_001091647.2), *Chelonia mydas* (XP_037736639.1), and *Coturnix japonica* (XP_015717693.1) were downloaded from the NCBI database (https://www.ncbi.nlm.nih.gov/gene, accessed on 6 November 2023). These sequences were used to analyze conserved motifs by the online software MEME 5.5.5 (https://meme-suite.org/meme/tools/meme, accessed on 6 November 2023).

The interaction of *lncLLM* and MYH9 protein were predicted by the online software catRAPID omics v2.0 (http://s.tartaglialab.com/, accessed on 6 November 2023).

### 4.7. Plasmid Construction and Small Interfering RNAs (siRNAs) Synthesis

For *lncLLM* overexpression plasmid construction, the full-length sequence of *lncLLM* was amplified by PCR, and we then cloned the expression plasmid pcDNA3.1 (Invitrogen, Carlsbad, CA, USA) by using NheI and HindIII restriction enzymes. The siRNA (si-*MYH9*) and lncRNA smart silencer (si-*lncLLM*) was synthesized from Ribobio (Guangzhou, China). si-*MYH9* was used to knockdown the *MYH9*, and then *lncLLM* smart silencer, which is a mixture of three siRNAs and three antisense oligonucleotides (ASOs), was used to knockdown *lncLLM* in cytoplasm and nucleus.

### 4.8. Cell Culture and Transfection

LMH cells were cultured in DMEM/F12 containing 10% fetal calf serum (FBS) (BI, Kibbutz, Beit Haemek, Israel), 25 U/mL penicillin, and 25 microg/mL streptomycin (Solarbio, Beijing, China) and cultured in an incubator containing 5% CO_2_ at 38 °C. Transfections were performed using Lipofectamine 3000 (Invitrogen, Carlsbad, CA, USA) according to the manufacturer’s instructions.

### 4.9. Protein Extraction and Western Blotting (WB) Assay

The cultured LMH cells were washed twice with phosphate buffer solution (PBS) (Solarbio, Beijing, China). Then, the total protein was extracted from cells by using radio immune precipitation assay (RIPA) reagent (Beyotime, Shanghai, China) containing 1% phenylmethyl sulfonyl fluoride (PMSF) (Servicebio, Wuhan, China) protease inhibitor. The protein concentrations were measured using a BCA kit (Beyotime, Shanghai, China) according to the manufacturer’s protocol.

The total protein after boiling denaturation was separated into 10% SDS-PAGE gels and transferred to polyvinylidene difluoride (PVDF) membranes (Millipore, Bedford, MA, USA). The membrane was immersed in a Tris-buffered saline-Tween 20 (TBST) solution containing 5% skim milk powder and blocked for 2 h. We then added the primary antibody and incubated overnight at 4 °C. Then, the membrane was washed thrice with TBST for 5 min and incubated with secondary antibody for 1 h at room temperature. Finally, the proteins were visualized using an ECL Western blotting detection kit (Beyotime, Shanghai, China) according to the manufacturer’s instructions. The authors used the following antibodies and dilutions: rabbit anti-MYH9 (1:5000, 11128-1-AP; Proteintech, Rosemont, IL, USA); rabbit anti-beta-actin (1:2000, 20536-1-AP; Proteintech).

### 4.10. FISH Assay and Co-Localization of lncRNA and Protein

FISH was performed using an RNA FISH Kit (RiboBio, Guangzhou, China). In brief, LMH cells cultured on coverslips were rinsed in PBS and fixed with 4% formaldehyde for 10 min. Then, the cells were permeabilized in PBS containing 0.5% Triton X-100 at 4 °C for 5 min, washed three times, and prehybridized at 37 °C for 30 min. Then, anti-*lncLLM*-AS1 oligodeoxynucleotide probes, designed and made by RiboBio (RiboBio, Guangzhou, China), diluted in hybridization solution were incubated with the cells in the dark at 37 °C overnight. The next day, the cells were stained with 4′, 6-diamidino-2-phenylindole (DAPI) and imaged using a fluorescence microscope (Carl Zeiss, Oberkochen, Germany). The anti-*lncLLM*-AS1 oligodeoxynucleotide probes is shown in Supplementary Table S3.

Immunofluorescence colocalization of *lncLLM* and MYH9 protein. The cells were fixed and permeabilized and then in situ hybridized with *lncLLM*-AS1 oligodeoxynucleotide probe. Afterwards, the cell chambers were incubated with anti-MYH9 (1:50, 11128-1-AP; Proteintech) overnight at 4 °C. The next day, after washing three times with PBS, the samples were incubated with Alexa Fluor 488-conjugated goat anti-rabbit secondary antibody (Thermo Fisher Scientific, Waltham, MA, USA) for 1 h at room temperature, washed with PBS, and then incubated with DAPI for 5 min, and the nuclei were analyzed. After staining, the cells were imaged under the above microscope.

### 4.11. Detection of Intracellular TG and TC

The intracellular content of TG and TC were detected using Tissue Triglyceride Assay kit and Tissue Total Cholesterol Assay kit (Applygen, Beijing, China), respectively, according to the manufacturer’s instructions. The protein concentrations were measured using a BCA kit (Applygen, Beijing, China) according to the manufacturer’s protocol to normalize TG and TC content.

### 4.12. Oil Red Stain

LMH cells cultured in 12-well plates and transfected for 24 h were washed twice with PBS fixed with 4% paraformaldehyde for 30 min and then stained with 2% oil red staining solution (Sigma-Aldrich, St. Louis, MO, USA) for 1 h. Subsequently, they were washed with PBS three times. An IX53 biological microscope (Olympus, Tokyo, Japan) was used to capture images of stained cells. After photographing, the cells were destained in 500 μL 100% isopropanol for 15 min and the oil red signal was quantified by measuring the absorbance at 510 nm (OD 510).

### 4.13. Cycloheximide (CHX) Chase Assay

CHX chase assay was performed to determine the half-life of MYH9. In brief, LMH cells were transfected with pcDNA3.1 and *lncLLM* or si-NC and the lncRNA smart silencer for 24 h. Subsequently, the cells were treated with medium containing 50 μg/mL cycloheximide (Meilunbio, Dalian, China) and 10% FBS to inhibit de novo protein translation. Cells were harvested at 0 h, 6 h, 12 h, and 18 h after CHX treatment for protein extraction. Western blotting was performed to detect protein expression.

### 4.14. Chromatin Isolation by RNA Purification-Mass Spectrometry (ChIRP-MS) Assay

CHIRP-MS assays were performed to explore the proteins that specifically bind to *lncLLM* and the interactions between them. LMH cells were washed with PBS and cross-linked with formaldehyde. Next, cells were hybridized with RNA antisense probes labeled with biotin and bound to magnetic beads. Sequences of RNA antisense probes designed for *lncLLM* are shown in Supplementary Table S4. After trypsin hydrolysis, peptide desalting, and other strong denaturing processes, the nonspecific binding proteins were removed and the RNA binding proteins (RBPs) were obtained. Thereafter, liquid chromatography tandem mass spectrometry (LC-MS/MS) was used to analyze the obtained RBPs.

### 4.15. RNA Immunoprecipitation (RIP) Assay

The RIP assay was performed with the Imprint RIP Kit (Sigma-Aldrich, St. Louis, MO, USA) using 5 μg of rabbit anti-MYH9 antibody (11128-1-AP; Proteintech) or rabbit IgG. RNAs that co-precipitated with MYH9 and IgG were extracted with Trizol reagent, and *lncLLM* enrichment was detected using qRT-PCR.

### 4.16. In Vivo Ubiquitination Assay

LMH cells were transfected with His-ubiquitin and *lncLLM* plasmids. Subsequently, 18 h after transfection, cells were treated with MG132 (10 µM) for 6 h. The whole-cell extracts prepared by lysis buffer were subjected to immunoprecipitation of Ni-NTA Agarose (#169045429; Qiagen, Hilden Germany). The levels of ubiquitinated protein were then detected by immunoblotting with MYH9 antibody (1:5000, 11128-1-AP; Proteintech).

### 4.17. Statistical Analysis

All experiment data are presented as the mean ± SEM. Statistical significance of differences between means was assessed by performing an unpaired Student’s *t*-test and *p* < 0.05 or less was considered significant. * means 0.01 < *p* < 0.05, ** *p* < 0.01. The graphs were drawn by using GraphPad Prism 8.0 software.

## 5. Conclusions

In this study we discovered a MYH9 protein regulator that promotes MYH9 protein ubiquitination. We explored the biological function of MYH9 in liver lipid metabolism and compared the conservation of MYH9 among species. This study improves laying hen performance and provides potential insights and molecular targets for the treatment of fatty liver disease in humans.

## Data Availability

The data presented in this study are available on request from the corresponding author.

## Supplementary Materials

The following supporting information can be downloaded at: https://www.mdpi.com/article/10.3390/ijms251910316/s1.
