# Supplementary material for: LncRNA lncLLM Facilitates Lipid Deposition by Promoting the Ubiquitination of MYH9 in Chicken LMH Cells
Source: Int J Mol Sci. 2024 Sep 25;25(19):10316. doi: 10.3390/ijms251910316 (PMC11477197; doi:10.3390/ijms251910316)
Supplement: Supplementary file 1 [file ijms-25-10316-s001.zip › ijms-3194699-supplementary.pdf]

Supplementary Table S1. The sequence of primers used for qRT-PCR.

Gene	Primer sequence (5' to 3')	
lncLLM	F:	CACAGCGCACTTACTGTGC
	R:	CGTGTTTGTGATCGCCTCAC
MYH9	F:	GTCACGCGCAATTCGTCAAG
	R:	GACCCGGGATCGTAACATGTC
FASN	F:	AGAGGCTTTGAAGCTCGGAC
	R:	GGTGCCTGAATACTTGGGCT
ACACA	F:	GCCTCCGAGAACCCAA
	R:	CCAGCAGTCTGAGCCACTA
SCD	F:	CAAGTTCTCCGAGACGCATG
	R:	GGGCTTGTAGTATCTCCGCT
AGPAT2	F:	CACCGTCAAGAACATGAGGA
	R:	ACCTCCATCAGCCCCATCAT
DGAT2	F:	ACTCCAAGCCCATCACCACT
	R:	CAACCCCGAACCTGCCTTTGT
PPARA	F:	AGGAGAACCATCCGATTGA
	R:	CTCAGACCTTGGCATTCGT
ATGL	F:	CTGAACCAAGCTCTTTTGGAG
	R:	ATATCTGGAAGCCATTCCAGC
CPT1	F:	CAAGCCAATTCTTCTGATGGG
	R:	TTCAACAGTCTGCCATCATGG
HMGCR	F:	GCGAGGAGTGTCTATTCGCA
	R:	ATAGTGGTCCTGCTACGCCT
SQLE	F:	CATCATGGGTCTCCGAAGGG
	R:	GCGGTGCATGAAGTTCCTTA
SREBP2	F:	CACCTGTGGAACAGCCTCAA
	R:	GGTGAGGCATGGTAGGTCTC
CYP7A1	F:	GATCTTCCCAGCCCTTGTGG
	R:	AGGAATGGTGTTAGCTTGCGA
CYP27A1	F:	GATGCTGCGGGCTGTTATCA
	R:	GTCTCGTCATGGGACATGGC
CYP450	F:	GGAAGCTACAGCATGGACGTAG
	R:	TGGTCAGCTTCTGCATCTCTC
MTTPL	F:	GATATCTCAGCCAACGTGGATG
	R:	CAGGGCTACTGGAAAATCTCAG
ApoB	F:	ATGTTCAAAAGATGCGGCCC
	R:	GCATGGCTCTTCTCTCACTG
apoVLDLII	F:	CAGGGCATTGGTGATAGCTG
	R:	CCAGCTCTAGGGGACACC

Note: F: indicates Forward primer; R: indicates Reverse primer.

Supplementary Table S2. The sequence of primers used for RACE PCR.

Primer name	Primer sequence (5’ to 3’)
lncLLM-3’RACE-outer	ATGTGAAATCTGCCTCCCAGCAGCATGG
lncLLM-3’RACE-inner	GAACAAGGTACAGGTGCCAACTGGCAGC
lncLLM-5’RACE-outer	GAGACAATTGGTCTGCAGGGATGAGGGG
lncLLM-5’RACE-inner	AGAGGTAACGCACCGAGAAGGACCAAGC

Supplementary Table S3. The probe sequence information of *lncLLM* for FISH

Probe number	Probe sequence (5' to 3')
anti-*lncLLM*-AS1	ACCCTCAAGGTCCTTAATGTCCGC
	ACAGAGGTAACGCACCGAGAAGGAC

Supplementary Table S4. The specific probes labeled with biotin were used to pull down the proteins that directly bind to *lncLLM*.

**Probe number**	**Probe sequence (5’ to 3’)**
lncLLM-P1	GACCACTTCCTGAAATGGGT
lncLLM-P2	GCTCACGTATCCTTCATGAC
lncLLM-P3	TGGATGGATGGATGGATGGA
lncLLM-P4	ATCTGCTGTCAATAACCCGT
lncLLM-P5	CACATTCTGAGGAAGATTTC
lncLLM-P6	CCTCCAAACACAGTTATCTC
lncLLM-P7	CCTCAACTCTGATACTGAAG
lncLLM-P8	CGTGTTTGTGATCGCCTCAC
lncLLM-P9	AGGGATGAGGGGTGGCACAG
lncLLM-P10	GACTAGGTTCCTTCTGCTGT
lncLLM-P11	GGTACCCTCGGTTCACCCTC
lncLLM-P12	CTCCCTGTCTCCTCGGAGCC
